# Prognostic value of esophageal cancer immune prognostic index in advanced esophageal squamous cell carcinoma patients with anti‐programmed cell death‐1 therapy

**DOI:** 10.1002/cam4.5844

**Published:** 2023-03-23

**Authors:** Jiangyue Lu, Lehui Du, Xiao Lei, Zhibo Zhang

**Affiliations:** ^1^ Department of Radiation Oncology The First Medical Center of Chinese PLA General Hospital Beijing China; ^2^ Medical School of Chinese PLA Beijing China; ^3^ Department of Radiation Oncology Chinese PLA General Hospital Beijing China; ^4^ The 78th Group Army Hospital of Chinese PLA Mudanjiang China

**Keywords:** efficacy, esophageal cancer immune prognostic index, esophageal squamous cell carcinoma, prognosis, programmed cell death‐1 inhibitor

## Abstract

**Background:**

This study aimed to determine whether the immune prognostic index (ECIPI), based on hemoglobin (Hb) and neutrophil‐to‐lymphocyte ratio (NLR), could predict the prognosis in patients with advanced esophageal squamous cell carcinoma (ESCC) receiving programmed cell death‐1 (PD‐1) inhibitor treatment.

**Methods:**

Advanced ESCC patients who had been treated with PD‐1 inhibitors from Jan 2016 to Oct 2021 were included. Kaplan–Meier method and Cox proportional hazards regression were used to analyze progression‐free survival (PFS) and overall survival (OS). The overall response rate (ORR) was the percentage of complete and partial responses. Univariate and multivariate analyses were used for estimating hazard ratio (HR) and 95% confidence interval (CI). Patients were grouped by ECIPI (good: Hb > 105 g/L and NLR ≤ 4.3; intermediate: Hb ≤ 105 g/L and NLR ≤ 4.3, or Hb > 105 g/L and NLR < 4.3; poor: Hb ≤ 105 g/L and NLR > 4.3). Variables for the multivariate model were selected if the *p*‐value was below 0.05 in the univariate analysis. All statistical comparisons were two‐way, and a *p*‐value below 0.05 was set as statistical significance.

**Results:**

Totally, of 123 ESCC patients with stage III or IV were included in the study. Efficacy evaluation showed that patients with pretreatment ECIPI good had the best ORR compared with those with ECIPI intermediate and ECIPI poor (53% vs. 22% vs. 8%, *p* < 0.01). Multivariate analysis showed that ECIPI was an independent influential factor for PFS (*p* = 0.004) and OS (*p* < 0.001). Kaplan–Meier curves demonstrated that patients with ECIPI good had the longest PFS (median: 11.6 vs. 3.5 vs. 1.7 months, *p* < 0.0001) and OS (median: 23.6 vs. 16.7 vs. 4.0 months, *p* < 0.0001) compared with those with ECIPI intermediate and ECIPI poor. Subgroup analysis indicated that ECIPI good was associated with improved PFS and OS in patients with ECOG 0–1, PD‐1 inhibitor plus chemotherapy, first‐line treatment, and smoke (all *p* < 0.05).

**Conclusions:**

Pretreatment ECIPI was associated with the prognosis in advanced ESCC patients with anti‐PD‐1 therapy, suggesting that ECIPI may be a useful tool to identify patients likely sensitive to PD‐1 inhibitors.

## Graphical abstract

Pretreatment ECIPI predicts the prognosis in advanced ESCC patiens treated with PD‐1 inhibitors.

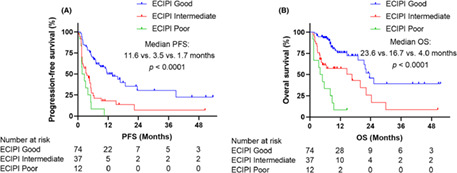

## 1 INTRODUCTION

Esophageal cancer (EC) ranks as the seventh most common cancer, and the sixth leading cause of cancer‐related death threat seriously to human health worldwide.^1^ Esophageal squamous cell carcinoma (ESCC) is the main histological subtype, which accounts for about 90% of EC.^2^, ^3^ For most ESCCs are metastatic at diagnosis, systemic chemotherapy remains the main treatment option with a 5‐year survival rate below 5%.^4^ Thus, new drugs and treatment strategies need to be developed to improve the therapeutic effect.

In recent years, immunotherapy has been recognized as an exciting therapeutic strategy in various cancers.^5^ By inhibiting the pathway of immune checkpoints, the immune system of patients can be activated to fight against tumor cells.^6^ Particularly, inhibition of programmed death‐1 (PD‐1) and programmed death‐ligand 1 (PD‐L1) have gained enormous clinical utility in a variety of malignancies including ESCC.^7^, ^8^ However, these benefits are limited to a small percentage of patients. Therefore, there is an urgent need to identify patients likely to respond to immunotherapy.

Anemia is a common hematologic abnormality in advanced cancer patients.^9^ Previous research showed that pretreatment hemoglobin (Hb) level was associated with the prognosis of non‐small cell lung cancer (NSCLC) patients receiving immunotherapy.^10^ Meanwhile, growing research has reported the key role of inflammation in the process of cancer initiation, development, and metastasis.^11^ The neutrophil‐to‐lymphocyte ratio (NLR), calculated by absolute neutrophil count (ANC) and absolute lymphocyte count (ALC), represents the inflammatory state of tumors and has been investigated in a variety of solid tumors including EC.^12^, ^13^, ^14^ However, no studies have yet described the predictive value of esophageal cancer immunological prognostic index (ECIPI, combining Hb and NLR) in advanced ESCC patients receiving immunotherapy. Therefore, this study aimed to investigate the association between pretreatment ECIPI and prognosis in advanced ESCC patients treated with PD‐1 inhibitors.

## 2 METHODS

### 2.1 Patients

Patients with ESCC who had been treated with PD‐1 inhibitors from Jan 2016 to Oct 2021 were identified in the Chinese PLA General Hospital. Patients should be excluded according to the following criteria: (I) patients received one cycle of PD‐1 inhibitor treatment; (II) baseline Hb, ANC, and ALC were not measured before initial immunotherapy (within 5 days); (III) treatment efficacy was not evaluated after initial immunotherapy (generally 6 weeks); (IV) patients with TNM stage I or II.

### 2.2 Data extraction and definition

Two independent investigators (J.Y. Lu and Z.B. Zhang) extracted data from medical records, including age, gender, clinical stage, smoking history, Eastern Cooperative Oncology Group performance status (ECOG PS), treatment strategy (PD‐1 inhibitor plus chemotherapy or radiotherapy), treatment line of immunotherapy (1‐line, 2‐line, and ≥ 3‐line), treatment response evaluation, blood results of Hb, ANC, and ALC at baseline. Disagreements were resolved through discussion with the other two investigators (X. Lei and L.H. Du).

The ECIPI was developed based on Hb and NLR, and the cutoff values of Hb and NLR were estimated by X‐tile v3.6.1.^15^ Patients were divided into three groups by ECIPI (good: Hb > 105 g/L and NLR ≤ 4.3; intermediate: Hb ≤ 105 g/L and NLR ≤ 4.3, or Hb > 105 g/L and NLR < 4.3; poor: Hb ≤ 105 g/L and NLR > 4.3). The Response Evaluation Criteria in Solid Tumors (v1.1) was used for evaluating treatment efficacy.^16^ Overall response rate (ORR) referred to the rate of complete response (CR) and partial response (PR). Progression‐free survival (PFS) was the interval from initiation of immunotherapy to disease progression, death, or the last follow‐up (if censored). Overall survival (OS) was the interval from the initiation of immunotherapy to death or the last follow‐up (if censored). All patients had been followed up by telephone and searching medical records until Jan 29, 2022.

### 2.3 Statistical analysis

All statistical analyses were performed using SPSS v23.0 and graphs were drawn using GraphPad Prism 8.0. The cutoff values for Hb and NLR were determined with X‐tile v3.6.1. Comparations between categorical variables were performed using Pearson's chi‐square test or Fisher's exact test. The Kaplan–Meier method was used for survival analysis and the log‐rank test for comparison. Cox proportional hazards regression models were used for identifying independently influential factors and estimating their hazard ratios (HR) and 95% confidence intervals (CI). Variables included in the multivariate model were selected if the *p‐*value of below 0.05 in the univariate analysis. All statistical comparisons were two‐way, and a *p‐*value of below 0.05 was set as statistical significance.

## 3 RESULTS

### 3.1 Patient selection and characteristics

**FIGURE 1:**
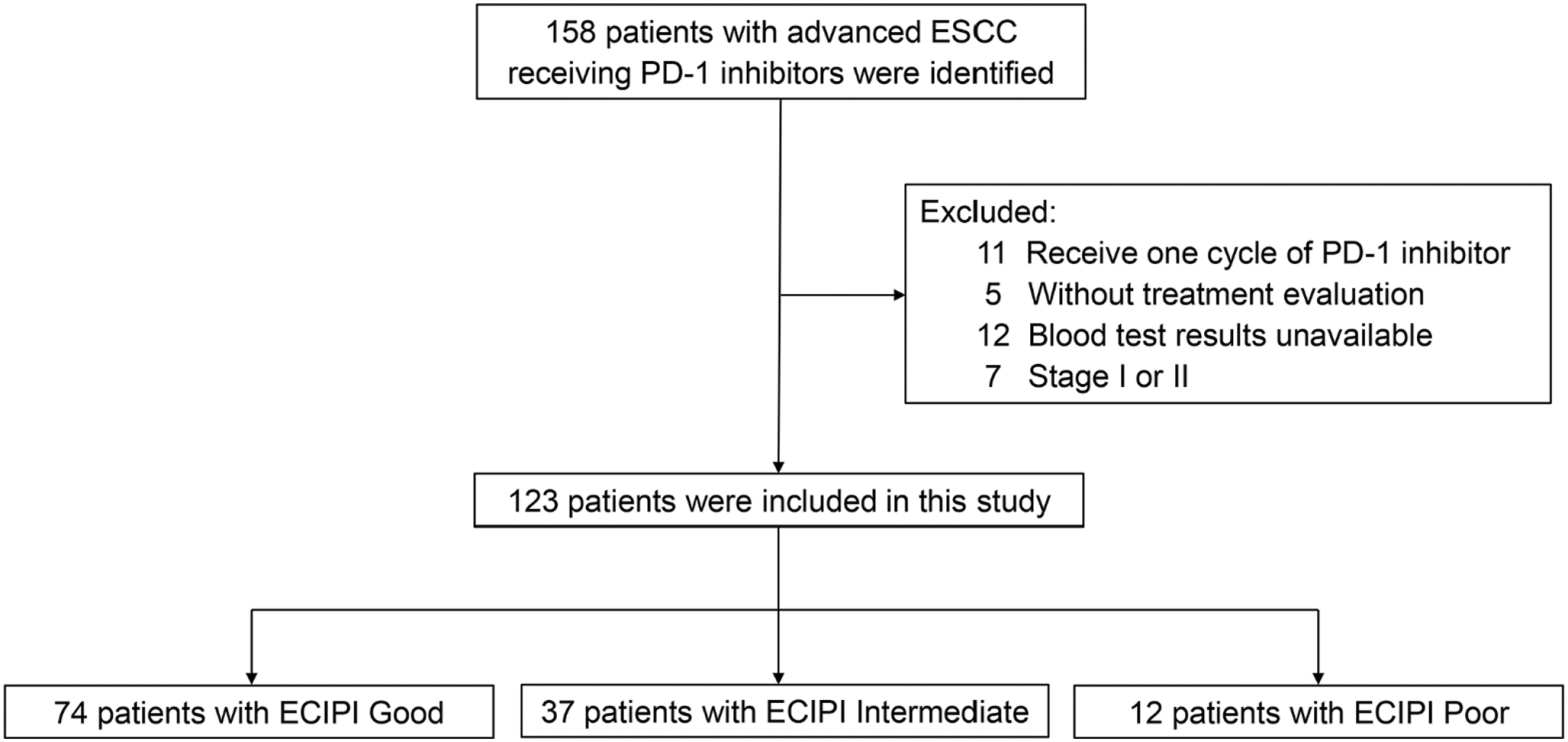
Flow chart of the study.

Initially, 158 ESCC patients treated with PD‐1 inhibitors were identified, of which, 35 patients were excluded including 11 patients with one cycle of immunotherapy, 5 patients without treatment assessment, 12 patients without blood results at baseline, 2 patients with stage I, and 5 patients with stage II. Ultimately, 123 patients were used for analysis in this study (Figure 1). Patient characteristics at baseline are shown in Table 1. Of all patients, the median follow‐up was 20.0 months and the median age was 61 years (range: 40–80); 91.1% were male, 82.9% with stage IV, and 66.7% were former smokers; most of the patients (92.7%) with ECOG 0–1. 73.2% received PD‐1 inhibitors plus chemotherapy, and 11.4% received radiotherapy; 52 patients (42.3%) received pembrolizumab treatment; first‐line treatment, second‐line treatment, and third‐line and above treatment accounted for 52.8%, 30.9%, and 16.3%, respectively; treatment efficacy of PR, stable disease (SD), and progressive disease (PD) accounted for 39.0%, 41.5%, and 19.5%, respectively; 21 patients (17.1%) had Hb values below 105 g/L, and 40 patients (32.5%) had NLR above 4.3. ECIPI good was in 74 patients (60.2%), intermediate in 37 patients (30.1%), and poor in 12 patients (9.8%), respectively. Until the last follow‐up, 43.1% of patients had died.

**TABLE 1:** Characteristics of the included patients.

Characteristics	No. of patients (*N* = 123)	Percentage (%)
Age (year), median (range)	61 (40–80)	
≤60	60	48.8
>60	63	51.2
Gender		
Male	112	91.1
Female	11	8.9
Stage		
III	21	17.1
IV	102	82.9
Smoking history		
Never smoke	41	33.3
Smoke	82	66.7
ECOG PS		
0–1	114	92.7
≥2	9	7.3
PD‐1 inhibitor plus chemotherapy		
Yes	90	73.2
No	33	26.8
PD‐1 inhibitor plus radiotherapy		
Yes	14	11.4
No	109	88.6
PD‐1 inhibitor		
Pembrolizumab	52	42.3
Nivolumab	24	19.5
Camrelizumab	7	5.6
Sintilimab	13	10.6
Toripalizumab	27	22.0
Treatment line		
First‐line	65	52.8
Second‐line	38	30.9
Third‐line and above	20	16.3
Treatment efficacy		
PR	48	39.0
SD	51	41.5
PD	24	19.5
Hb (g/L)		
Median (range)	126 (79–170)	
≤105	21	17.1
>105	102	82.9
NLR		
Median (range)	3.2 (0.9–89.7)	
≤4.3	83	67.5
>4.3	40	32.5
ECIPI		
Good	74	60.2
Intermediate	37	30.1
Poor	12	9.8

Abbreviations: ECIPI, esophageal cancer immune prognostic index; ECOG PS, Eastern Cooperative Oncology Group performance status; ESCC, esophageal squamous cell carcinoma; Hb, hemoglobin; NLR, neutrophil‐to‐lymphocyte ratio; PD, progressive disease; PD‐1, programmed cell death‐1; PR, partial response; SD, steady disease.

### 3.2 Univariate and multivariate analyses of PFS and OS for ECIPI

Variables of ECOG PS, combine chemotherapy, treatment line, and ECIPI were associated with PFS in univariate analysis (*p* < 0.05), and multivariate analysis revealed that ECOG 0–1 (HR: 0.25, 95% CI: 0.11–0.56, *p* = 0.001), first‐line treatment (HR: 0.42, 95% CI: 0.20–0.85, *p* = 0.016), and ECIPI good (HR: 0.32, 95% CI: 0.11–0.92, *p* = 0.034) were independently associated with prolonged PFS in comparison with ECOG ≥ 2, third‐line and above treatment, and ECIPI poor, respectively (Table 2). Variables of ECOG PS, combine chemotherapy, treatment line, and ECIPI were correlated with OS in univariate analysis (*p* < 0.05), after multivariate analysis, the results showed that ECOG 0–1 (HR: 0.08, 95% CI: 0.03–0.21, *p* < 0.001) and ECIPI good (HR: 0.12, 95% CI: 0.05–0.31, *p* < 0.001) or ECIPI intermediate (HR: 0.30, 95% CI: 0.12–0.72, *p* = 0.007) were independently correlated with improved OS in comparison with ECOG ≥ 2 and ECIPI poor (Table 3).

**TABLE 2:** Univariate and multivariate analyses for PFS.

Variable	Category	Univariate analysis		Multivariate analysis	
		HR (95% CI)	*p‐value*	HR (95% CI)	*p*‐value
Age (year)	>60 vs. ≤60	0.73 (0.47, 1.13)	0.157	—	—
Gender	Female vs. Male	0.94 (0.45, 1.95)	0.865	—	—
Smoking history	Yes vs. No	0.75 (0.48, 1.17)	0.205	—	—
ECOG PS	0–1 vs. ≥2	0.15 (0.07, 0.32)	<0.001***	0.25 (0.11, 0.55)	0.001***
PD‐1 inhibitor plus chemotherapy	Yes vs. No	0.42 (0.26, 0.66)	<0.001***	0.73 (0.43, 1.25)	0.255
PD‐1 inhibitor plus radiotherapy	Yes vs. No	0.46 (0.21, 1.01)	0.052	—	—
Treatment line			<0.001***		0.048
	First‐line vs. ≥ Third‐line	0.23 (0.13, 0.42)	<0.001***	0.42 (0.20, 0.85)	0.016*
	Second‐line vs. ≥ Third‐line	0.70 (0.340, 1.24)	0.224	0.75 (0.41, 1.35)	0.330
ECIPI			<0.001***		0.004***
	Good vs. Poor	0.19 (0.10, 0.37)	<0.001***	0.32 (0.11, 0.92)	0.034*
	Intermediate vs. Poor	0.53 (0.27, 1.04)	0.066	0.79 (0.31, 1.97)	0.609
Stage	IV vs. III	0.76 (0.54, 1.05)	0.096	—	—

Abbreviations: CI, confidence interval; ECIPI, esophageal cancer immune prognostic index; ECOG PS, Eastern Cooperative Oncology Group performance status; HR, hazard ratio; PFS, progression‐free survival.

* *p* < 0.05

*** *p* < 0.001.

**TABLE 3:** Univariate and multivariate analyses for OS.

Variable	Category	Univariate analysis		Multivariate analysis	
		HR (95% CI)	*p*‐value	HR (95% CI)	*p*‐value
Age (year)	>60 vs. ≤60	0.78 (0.46, 1.34)	0.374	—	—
Gender	Female vs. Male	0.46 (0.14, 1.49)	0.198	—	—
Smoking history	Yes vs. No	0.89 (0.50, 1.57)	0.688	—	—
ECOG PS	0–1 vs. ≥2	0.06 (0.03, 0.14)	<0.001***	0.08 (0.03, 0.21)	<0.001***
PD‐1 inhibitor plus chemotherapy	Yes vs. No	0.41 (0.24, 0.71)	0.002**	0.57 (0.29, 1.13)	0.109
PD‐1 inhibitor plus radiotherapy	Yes vs. No	0.13 (0.02, 0.94)	0.004**	0.27 (0.04, 2.04)	0.206
Treatment line			<0.001***		0.616
	First‐line vs. ≥ Third‐line	0.21 (0.10, 0.42)	<0.001***	0.74 (0.31, 1.76)	0.489
	Second‐line vs. ≥ Third‐line	0.55 (0.28, 1.08)	0.083	0.71 (0.35, 1.43)	0.335
ECIPI			<0.001***		<0.001***
	Good vs. Poor	0.12 (9,96, 9,26)	<0.001***	0.12 (0.05, 0.31)	<0.001***
	Intermediate vs. Poor	0.34 (0.16, 0.73)	0.005***	0.30 (0.12, 0.72)	0.007***
Stage	IV vs. III	1.01 (0.71, 1.45)	0.961	—	—

Abbreviations: ECOG PS, Eastern Cooperative Oncology Group performance status; ECIPI, esophageal cancer immune prognostic index; HR, hazard ratio; CI, confidence interval; OS, overall survival.

** *p* < 0.01

*** *p* < 0.001.

### 3.3 Treatment efficacy and survival analysis for ECIPI

**FIGURE 2:**
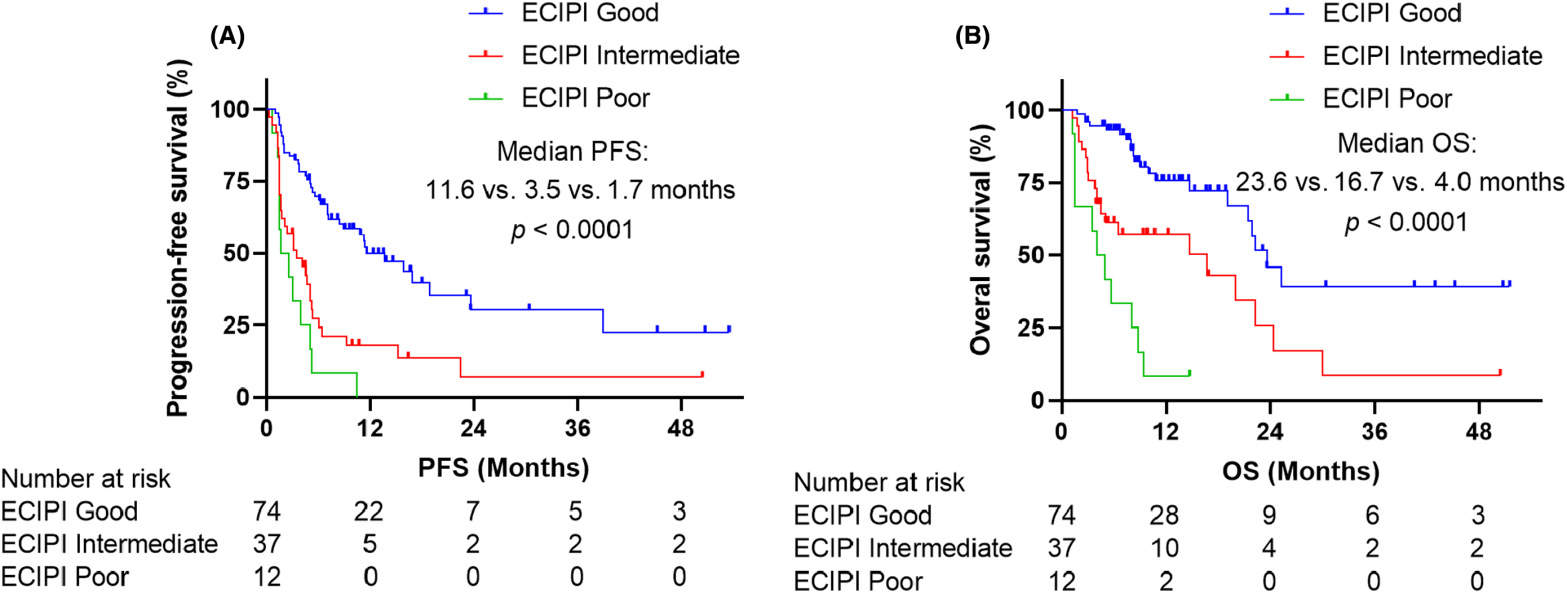
Survival analysis among the three ECIPI groups. (A) comparing PFS among the three ECIPI groups; (B) comparing OS among the three ECIPI groups. ECIPI, esophageal cancer immune prognostic index; OS, overall survival; PFS, progression‐free survival.

As shown in Table 4, treatment efficacy analysis showed that patients with ECIPI good had the best ORR compared with those with ECIPI intermediate and ECIPI poor (53% vs. 22% vs. 8%, *p* < 0.001). Kaplan–Meier survival curves demonstrated that patients with ECIPI good had the longest PFS (median: 11.6 vs. 3.5 vs. 1.7 months, *p* < 0.0001) and OS (median: 23.6 vs. 16.7 vs. 4.0 months, *p* < 0.0001) compared with those with ECIPI intermediate and ECIPI poor (Figure 2).

**TABLE 4:** Treatment efficacy among the three ECIPI groups.

Treatment efficacy	ECIPI			
	Good	Intermediate	Poor	*p*‐value
PR	39	8	1	0.003**
SD	25	19	7	—
PD	10	10	4	—
ORR	0.53	0.22	0.08	<0.001***

Abbreviations: ECIPI, esophageal cancer immune prognostic index; ORR, overall response rate; PD, progressive disease; PR, partial response; SD, steady disease.

** *p* < 0.01

*** *p* < 0.001.

### 3.4 Subgroup analysis for ECIPI

**FIGURE 3:**
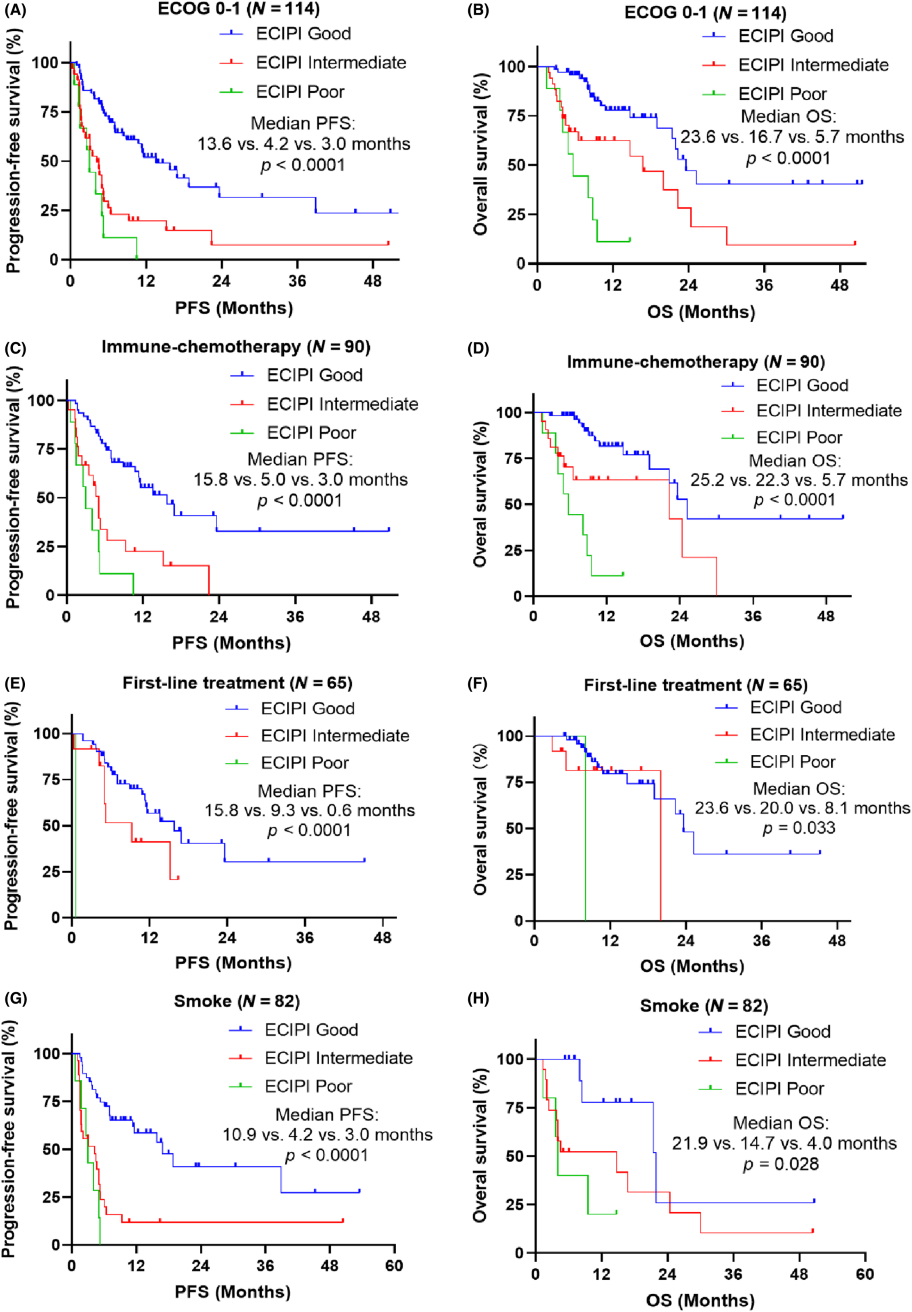
Subgroup analysis of PFS and OS. (A) comparing PFS in ECOG 0–1 subgroup; (B) comparing OS in ECOG 0–1 subgroup; (C) comparing PFS in immune‐chemotherapy subgroup; (D) comparing OS in immune‐chemotherapy subgroup; (E) comparing PFS in first‐line treatment subgroup; (F) comparing OS in first‐line treatment subgroup; (G) comparing PFS in smoke subgroup; (H) comparing OS in smoke subgroup. ECIPI, esophageal cancer immune prognostic index; ECOG, Eastern Cooperative Oncology Group; OS, overall survival; PFS, progression‐free survival.

The differences in characteristics among the three ECIPI groups are shown in Table 5. Variables of ECOG PS, treatment strategy, and treatment line were unbalanced among the three ECIPI groups (*p* < 0.05). The proportion of ECOG 0–1 (62% vs. 33%, *p* = 0.035), PD‐1 inhibitor plus chemotherapy (67% vs. 42%, *p* = 0.024), and first‐line treatment (80% vs. 37% vs. 40%, *p* < 0.001) were higher in patients with ECIPI good than that of ECOG ≥ 2, without chemotherapy, second‐line treatment, and third‐line and above treatment. Subgroup analysis showed that ECIPI good was associated with improved PFS and OS in patients with ECOG 0–1, PD‐1 inhibitor plus chemotherapy, first‐line treatment, and smoke with all *p* < 0.05 (Figure 3).

**TABLE 5:** Comparisons of characteristics among the three ECIPI groups.

Characteristics	ECIPI			*X* ^*2*^	*p*‐value
	Good	Intermediate	Poor		
Age					
≤60	31	22	7	3.533	0.171
>60	43	15	5		
Gender					
Male	64	37	11	5.538	0.063
Female	10	0	1		
Stage					
III	15	4	2	1.561	0.458
IV	59	33	10		
Smoking history					
Never smoke	26	10	5	1.145	0.564
Smoke	48	27	7		
ECOG PS					
0–1	71	34	9	6.729	0.035*
≥ 2	3	3	3		
PD‐1 inhibitor plus chemotherapy					
Yes	60	21	9	7.457	0.024*
No	14	16	3		
PD‐1 inhibitor plus radiotherapy					
Yes	12	2	0	4.566	0.102
No	62	35	12		
Treatment line					
First‐line	52	12	1	30.319	<0.001***
Second‐line	14	19	5		
Third‐line and above	8	6	6		

Abbreviations: ECIPI, esophageal cancer immune prognostic index; ECOG PS, Eastern Cooperative Oncology Group performance status; ESCC, esophageal squamous cell carcinoma.

* *p* < 0.05

*** *p* < 0.001.

## 4 DISCUSSION

Though EC is one of the most lethal cancers worldwide, its treatment development has made modest improvements in recent years.^17^ ESCC accounts for the majority of EC and is a frustrating disease with limited treatment options. The clinical application of anti‐PD‐1 therapy has revolutionized cancer treatment. PD‐1/PD‐L1 targeted immunotherapy is considered an innovative treatment strategy with durable anticancer activity and long survival,^18^ however, the effectiveness of immunotherapy is below 20%, and most patients cannot experience treatment benefits.^19^, ^20^ Although the incidence of immune‐related adverse events caused by immunotherapy was low, some were serious and even life‐threatening.^21^, ^22^, ^23^, ^24^ Therefore, there is a necessity to investigate biomarkers to identify the most suitable patients for such therapy. Biomarkers such as PD‐L1 expression and tumor mutational burden were clinically used predictors, but the detection of these biomarkers requires assays and their predictive values are unsatisfactory.^25^, ^26^ Convenient and practical biomarker identification is a necessary guarantee for clinical practice.

Anemia is one of the most common hematological abnormalities in cancers, and its incidence increases with the progression of malignancy and the intensification of cancer treatment.^9^ Hemoglobin below 110 g/L is considered the diagnostic of cancer‐related hemoglobin loss.^27^ Previous research reported that anemia in patients with advanced cancer leads to insufficient T‐cell responses and induces immunosuppression.^28^ In addition, hypoxia induced by Hb reduction may stimulate tumor growth and progression, reduce tumor sensitivity to cancer therapy, and ultimately lead to poor survival.^29^, ^30^ Clinical research showed that pretreatment low Hb level was correlated with poor prognosis in cancer patients.^31^, ^32^, ^33^ However, several studies reported this association was not significant.^34^, ^35^ Currently, no studies have evaluated the predictive role of Hb in ESCC patients with PD‐1 inhibitor treatment. Our results suggested that patients with pretreatment low Hb were associated with worse clinical outcomes. Decreased Hb was an independent factor for shortened OS in multivariate analysis, but only a tendency to shorten PFS was observed for the small sample size and interactions of other influencing factors.

Inflammation has a critical value in the occurrence, development, and metastasis of cancer, and increasing evidence link inflammation to the prognosis of patients with cancer.^36^, ^37^, ^38^ Elevated neutrophils reflect systemic and local inflammatory responses. Neutrophils provide a favorable microenvironment for tumor cell proliferation and promote the progression and invasion of tumor cells.^11^ In contrast, a reduced number of lymphocytes could suppress the immune response to cancer.^39^, ^40^ The NLR, calculated based on neutrophils and lymphocytes, has emerged as a potential cancer prognostic biomarker of clinical importance, especially because of its availability and the fact that this relationship can be easily calculated from a patient's routine blood count. Although previous studies investigated the role of NLR, especially its association with the prognosis in various cancers,^41^, ^42^, ^43^, ^44^ it has not yet been well evaluated in patients with advanced ESCC with anti‐PD‐1 therapy. Our findings showed that NLR was an independent influential factor for PFS and OS in multivariate analysis, and patients with pretreatment low NLR could have better PFS and OS than those with high NLR.

The ECIPI was developed by combining Hb and NLR. We first evaluated the relationship between pretreatment ECIPI and the prognosis in patients with advanced ESCC receiving PD‐1 inhibitors. Our findings indicated that patients with ECIPI good had the best improvements in ORR, PFS, and OS, while those with ECIPI poor had the worst treatment outcomes. Pretreatment ECIPI may be a convenient and useful tool to help identify patients likely to get benefit from PD‐1 inhibitor treatment and those who may not obtain treatment benefits.

Some limitations should be considered in this study. First, this study may have some limitations for a lack of clinical data and selection bias. Second, there are other prognostic factors affecting Hb and NLR levels, such as infectious diseases. Our retrospective study failed to stratify infectious diseases, and the impact of NLR and Hb on the prognosis of ESCC patients receiving immunotherapy requires further study. Third, the optimal cut‐off values of Hb and NLR are not yet clear, we used X‐tile software to calculate the cutoff values based on our data, and the results showed that pretreatment Hb and NLR thresholds of 105 g/L and 4.3 were independently related to treatment outcomes. Finally, although some characteristics, such as ECOG PS, treatment strategy, and treatment line, may influence the final results, we performed a subgroup analysis and the results were still significant. Nevertheless, this study provides a useful and convenient tool to identify patients likely to respond to PD‐1 inhibitors and those who may be resistant to these drugs. Further research is warranted to clarify these findings.

## 5 CONCLUSION

Pretreatment ECIPI was correlated with the prognosis in advanced ESCC patients treated with PD‐1 inhibitors, suggesting that ECIPI is a useful and convenient tool for identifying patients who may benefit from PD‐1 inhibitors.

## AUTHOR CONTRIBUTIONS

**Jiangyue Lu:** Writing – original draft (lead); writing – review and editing (equal). **Lehui Du:** Writing – review and editing (equal). **Xiao Lei:** Writing – review and editing (equal). **Zhibo Zhang:** Conceptualization (lead); formal analysis (lead).

## FUNDING INFORMATION

None.

## CONFLICT OF INTEREST STATEMENT

All authors declare no conflicts of interest in this study.

## ETHICS APPROVAL

This study was approved by the Ethics Committee of Chinese PLA General Hospital, and informed consent was not required because the study was retrospective.

## Data Availability

The datasets generated for this study are available on request to the corresponding author.

## References

[1] Sung H , Ferlay J , Siegel RL , et al. Global cancer statistics 2020: globocan estimates of incidence and mortality worldwide for 36 cancers in 185 countries. CA Cancer J Clin. 2021;71(3):209‐249. 33538338 10.3322/caac.21660

[2] Lu CL , Lang HC , Luo JC , et al. Increasing trend of the incidence of esophageal squamous cell carcinoma, but not adenocarcinoma, in Taiwan. Cancer Causes Control. 2010;21(2):269‐274. 19866363 10.1007/s10552-009-9458-0

[3] Zhang HZ , Jin GF , Shen HB . Epidemiologic differences in esophageal cancer between Asian and Western populations. Chin J Cancer. 2012;31(6):281‐286. 22507220 10.5732/cjc.011.10390 PMC3777490

[4] Siegel RL , Miller KD , Fuchs HE , Jemal A . Cancer statistics, 2021. CA Cancer J Clin. 2021;71(1):7‐33. 33433946 10.3322/caac.21654

[5] Ribas A , Wolchok JD . Cancer immunotherapy using checkpoint blockade. Science. 2018;359(6382):1350‐1355. 29567705 10.1126/science.aar4060 PMC7391259

[6] Joyce JA , Fearon DT . T cell exclusion, immune privilege, and the tumor microenvironment. Science. 2015;348(6230):74‐80. 25838376 10.1126/science.aaa6204

[7] Janjigian YY , Bendell J , Calvo E , et al. CheckMate‐032 study: efficacy and safety of nivolumab and nivolumab plus ipilimumab in patients with metastatic esophagogastric cancer. J Clin Oncol. 2018;36(28):2836‐2844. 30110194 10.1200/JCO.2017.76.6212 PMC6161834

[8] Kudo T , Hamamoto Y , Kato K , et al. Nivolumab treatment for oesophageal squamous‐cell carcinoma: an open‐label, multicentre, phase 2 trial. Lancet Oncol. 2017;18(5):631‐639. 28314688 10.1016/S1470-2045(17)30181-X

[9] Ibrahim UA , Yusuf AA , Ahmed SG . The pathophysiologic basis of anaemia in patients with malignant diseases. Gulf J Oncolog. 2016;1(22):80‐89. 28191814

[10] Zhang Z , Zhang F , Yuan F , et al. Pretreatment hemoglobin level as a predictor to evaluate the efficacy of immune checkpoint inhibitors in patients with advanced non‐small cell lung cancer. Ther Adv Med Oncol. 2020;12:1758835920970049. 33224276 10.1177/1758835920970049 PMC7649885

[11] Grivennikov SI , Greten FR , Karin M . Immunity, inflammation, and cancer. Cell. 2010;140(6):883‐899. 20303878 10.1016/j.cell.2010.01.025 PMC2866629

[12] Gao Y , Zhang Z , Li Y , et al. Pretreatment neutrophil‐to‐lymphocyte ratio as a prognostic biomarker in unresectable or metastatic esophageal cancer patients with anti‐PD‐1 therapy. Front Oncol. 2022;12:834564. 35494073 10.3389/fonc.2022.834564 PMC9043597

[13] Chen S , Li R , Zhang Z , et al. Prognostic value of baseline and change in neutrophil‐to‐lymphocyte ratio for survival in advanced non‐small cell lung cancer patients with poor performance status receiving PD‐1 inhibitors. Transl Lung Cancer Res. 2021;10(3):1397‐1407. 33889518 10.21037/tlcr-21-43 PMC8044483

[14] Li Y , Zhang Z , Hu Y , et al. Pretreatment neutrophil‐to‐lymphocyte ratio (NLR) may predict the outcomes of advanced non‐small‐cell lung cancer (NSCLC) patients treated with immune checkpoint inhibitors (ICIs). Front Oncol. 2020;10:654. 32656072 10.3389/fonc.2020.00654 PMC7324627

[15] Camp RL , Dolled‐Filhart M , Rimm DL . X‐tile: a new bio‐informatics tool for biomarker assessment and outcome‐based cut‐point optimization. Clin Cancer Res. 2004;10(21):7252‐7259. 15534099 10.1158/1078-0432.CCR-04-0713

[16] Eisenhauer EA , Therasse P , Bogaerts J , et al. New response evaluation criteria in solid tumours: revised RECIST guideline (version 1.1). Eur J Cancer. 2009;45(2):228‐247. 19097774 10.1016/j.ejca.2008.10.026

[17] Baba Y , Yoshida N , Kinoshita K , et al. Clinical and prognostic features of patients with esophageal cancer and multiple primary cancers: a retrospective single‐institution study. Ann Surg. 2018;267(3):478‐483. 28151796 10.1097/SLA.0000000000002118

[18] Betof Warner A , Palmer JS , Shoushtari AN , et al. Long‐term outcomes and responses to retreatment in patients with melanoma treated with PD‐1 blockade. J Clin Oncol. 2020;38(15):1655‐1663. 32053428 10.1200/JCO.19.01464 PMC7238490

[19] Gettinger S , Horn L , Jackman D , et al. Five‐year follow‐up of nivolumab in previously treated advanced non‐small‐cell lung cancer: results from the CA209‐003 study. J Clin Oncol. 2018;36(17):1675‐1684. 29570421 10.1200/JCO.2017.77.0412

[20] Vokes EE , Ready N , Felip E , et al. Nivolumab versus docetaxel in previously treated advanced non‐small‐cell lung cancer (CheckMate 017 and CheckMate 057): 3‐year update and outcomes in patients with liver metastases. Ann Oncol. 2018;29(4):959‐965. 29408986 10.1093/annonc/mdy041

[21] Thompson JA , Schneider BJ , Brahmer J , et al. Management of immunotherapy‐related toxicities, version 1.2022, NCCN clinical practice guidelines in oncology. J Natl Compr Cancer Netw. 2022;20(4):387‐405. 10.6004/jnccn.2022.0020 35390769

[22] Haanen J , Obeid M , Spain L , et al. Management of toxicities from immunotherapy: ESMO clinical practice guideline for diagnosis, treatment and follow‐up. Ann Oncol. 2022;33:1217‐1238. 36270461 10.1016/j.annonc.2022.10.001

[23] Schneider BJ , Naidoo J , Santomasso BD , et al. Management of immune‐related adverse events in patients treated with immune checkpoint inhibitor therapy: ASCO guideline update. J Clin Oncol. 2021;39(36):4073‐4126. 34724392 10.1200/JCO.21.01440

[24] Brahmer JR , Abu‐Sbeih H , Ascierto PA , et al. Society for Immunotherapy of Cancer (SITC) clinical practice guideline on immune checkpoint inhibitor‐related adverse events. J Immunother Cancer. 2021;9(6):e002435. 34172516 10.1136/jitc-2021-002435 PMC8237720

[25] McLaughlin J , Han G , Schalper KA , et al. Quantitative assessment of the heterogeneity of PD‐L1 expression in non‐small‐cell lung cancer. JAMA Oncol. 2016;2(1):46‐54. 26562159 10.1001/jamaoncol.2015.3638 PMC4941982

[26] Chan TA , Yarchoan M , Jaffee E , et al. Development of tumor mutation burden as an immunotherapy biomarker: utility for the oncology clinic. Ann Oncol. 2019;30(1):44‐56. 30395155 10.1093/annonc/mdy495 PMC6336005

[27] Rodgers GM III , Becker PS , Blinder M , et al. NCCN clinical practice guidelines in oncology, cancer‐ and chemotherapy‐induced anemia (v.2.2011). J Natl Compr Canc Netw. 2012;10:628‐653. 22570293 10.6004/jnccn.2012.0064

[28] Zhao L , He R , Long H , et al. Late‐stage tumors induce anemia and immunosuppressive extramedullary erythroid progenitor cells. Nat Med. 2018;24(10):1536‐1544. 30297899 10.1038/s41591-018-0205-5 PMC6211844

[29] Höckel M , Vaupel P . Tumor hypoxia: definitions and current clinical, biologic, and molecular aspects. J Natl Cancer Inst. 2001;93(4):266‐276. 11181773 10.1093/jnci/93.4.266

[30] Tas F , Eralp Y , Basaran M , et al. Anemia in oncology practice: relation to diseases and their therapies. Am J Clin Oncol. 2002;25(4):371‐379. 12151968 10.1097/00000421-200208000-00011

[31] Huang Y , Wei S , Jiang N , et al. The prognostic impact of decreased pretreatment hemoglobin level on the survival of patients with lung cancer: a systematic review and meta‐analysis. BMC Cancer. 2018;18(1):1235. 30526532 10.1186/s12885-018-5136-5 PMC6288911

[32] Li J , Chen S , Peng S , et al. Prognostic nomogram for patients with nasopharyngeal carcinoma incorporating hematological biomarkers and clinical characteristics. Int J Biol Sci. 2018;14(5):549‐556. 29805306 10.7150/ijbs.24374 PMC5968847

[33] Caro JJ , Salas M , Ward A , Goss G . Anemia as an independent prognostic factor for survival in patients with cancer: a systemic, quantitative review. Cancer. 2001;91(12):2214‐2221. 11413508

[34] Garrido P , Rosell R , Massutí B , et al. Predictors of long‐term survival in patients with lung cancer included in the randomized Spanish lung cancer group 0008 phase II trial using concomitant chemoradiation with docetaxel and carboplatin plus induction or consolidation chemotherapy. Clin Lung Cancer. 2009;10(3):180‐186. 19443338 10.3816/CLC.2009.n.025

[35] Kishida Y , Hirose T , Shirai T , et al. Myelosuppression induced by concurrent chemoradiotherapy as a prognostic factor for patients with locally advanced non‐small cell lung cancer. Oncol Lett. 2011;2(5):949‐955. 22866156 10.3892/ol.2011.348 PMC3408010

[36] McMillan DC . The systemic inflammation‐based Glasgow Prognostic Score: a decade of experience in patients with cancer. Cancer Treat Rev. 2013;39(5):534‐540. 22995477 10.1016/j.ctrv.2012.08.003

[37] Coussens LM , Werb Z . Inflammation and cancer. Nature. 2002;420(6917):860‐867. 12490959 10.1038/nature01322 PMC2803035

[38] Rollins BJ . Inflammatory chemokines in cancer growth and progression. Eur J Cancer. 2006;42(6):760‐767. 16510278 10.1016/j.ejca.2006.01.002

[39] Song MK , Chung JS , Seol YM , et al. Influence of low absolute lymphocyte count of patients with nongerminal center type diffuse large B‐cell lymphoma with R‐CHOP therapy. Ann Oncol. 2010;21(1):140‐144. 19887468 10.1093/annonc/mdp505

[40] Avci N , Deligonul A , Tolunay S , et al. Prognostic impact of tumor lymphocytic infiltrates in patients with breast cancer undergoing neoadjuvant chemotherapy. J BUON. 2015;20(4):994‐1000. 26416041

[41] Templeton AJ , McNamara MG , Šeruga B , et al. Prognostic role of neutrophil‐to‐lymphocyte ratio in solid tumors: a systematic review and meta‐analysis. J Natl Cancer Inst. 2014;106(6):dju124. 24875653 10.1093/jnci/dju124

[42] Malietzis G , Giacometti M , Kennedy RH , Athanasiou T , Aziz O , Jenkins JT . The emerging role of neutrophil to lymphocyte ratio in determining colorectal cancer treatment outcomes: a systematic review and meta‐analysis. Ann Surg Oncol. 2014;21(12):3938‐3946. 24866438 10.1245/s10434-014-3815-2

[43] Yin Y , Wang J , Wang X , et al. Prognostic value of the neutrophil to lymphocyte ratio in lung cancer: a meta‐analysis. Clinics (Sao Paulo). 2015;70(7):524‐530. 26222823 10.6061/clinics/2015(07)10 PMC4498150

[44] Sun J , Chen X , Gao P , et al. Can the neutrophil to lymphocyte ratio Be used to determine gastric cancer treatment outcomes? A systematic review and meta‐analysis. Dis Markers. 2016;2016:7862469. 26924872 10.1155/2016/7862469 PMC4746375

